# Subsidized health insurance coverage of people in the informal sector and vulnerable population groups: trends in institutional design in Asia

**DOI:** 10.1186/s12939-016-0436-3

**Published:** 2016-10-04

**Authors:** Ileana Vilcu, Lilli Probst, Bayarsaikhan Dorjsuren, Inke Mathauer

**Affiliations:** 1 Department of Health Systems Governance and Financing, World Health Organization, Avenue Appia, Geneva, 1211 Switzerland; 2 Department of Health Systems Governance and Financing, World Health Organization, Avenue Appia, Geneva, 1211 Switzerland; 3 Department of Health Systems Governance and Financing, World Health Organization, Avenue Appia, Geneva, 1211 Switzerland

**Keywords:** Universal health coverage, Vulnerable population groups, Government subsidization of health insurance, Financial protection

## Abstract

**Background:**

Many low- and middle-income countries with a social health insurance system face challenges on their road towards universal health coverage (UHC), especially for people in the informal sector and vulnerable population groups or the informally employed. One way to address this is to subsidize their contributions through general government revenue transfers to the health insurance fund.

This paper provides an overview of such health financing arrangements in Asian low- and middle-income countries. The purpose is to assess the institutional design features of government subsidized health insurance type arrangements for vulnerable and informally employed population groups and to explore how these features contribute to UHC progress.

**Methods:**

This regional study is based on a literature search to collect country information on the specific institutional design features of such subsidization arrangements and data related to UHC progress indicators, i.e. population coverage, financial protection and access to care. The institutional design analysis focuses on eligibility rules, targeting and enrolment procedures; financing arrangements; the pooling architecture; and benefit entitlements.

**Results:**

Such financing arrangements currently exist in 8 countries with a total of 14 subsidization schemes. The most frequent groups covered are the poor, older persons and children. Membership in these arrangements is mostly mandatory as is full subsidization. An integrated pool for both the subsidized and the contributors exists in half of the countries, which is one of the most decisive features for equitable access and financial protection. Nonetheless, in most schemes, utilization rates of the subsidized are higher compared to the uninsured, but still lower compared to insured formal sector employees. Total population coverage rates, as well as a higher share of the subsidized in the total insured population are related with broader eligibility criteria.

**Conclusions:**

Overall, government subsidized health insurance type arrangements can be effective mechanism to help countries progress towards UHC, yet there is potential to improve on institutional design features as well as implementation.

**Electronic supplementary material:**

The online version of this article (doi:10.1186/s12939-016-0436-3) contains supplementary material, which is available to authorized users.

## Background

More and more countries engage in health financing reforms in order to progress towards universal health coverage (UHC) [1] along their country context and objectives. This serves to increase access for all to needed health services of good quality without facing financial hardship when seeking care [2]. Improved coverage with health services has been shown to lead to better health indicators and contribute to stronger economic development; at the same time, there are political benefits for political leaders who support a UHC agenda, “for the simple reason that the majority of people (and of the electorate) wants access to affordable, good quality health services” [3].

While not the only path towards UHC, an increasing number of low- and middle-income countries have established health insurance systems [4, 5]. Yet, the process of transition to UHC in many of these countries has proven to be challenging, particularly as to the coverage of people in the informal sector and other vulnerable population groups [2, 5–8]. Especially vulnerable and poor population groups cannot afford to pay contributions on their own, because they have no income, a very low income or a very unsteady income. One way to address this specific challenge, equally faced at some point by the (now high-income) countries, is government subsidization of contribution payments through the transfer of general government revenues to the health insurance fund [9]. Alternative terms and conceptualization include “premium subsidization” or “exemption from contributions”.

This health financing mechanism has a number of institutional characteristics of health insurance schemes and/or is built upon its logic of pooling health risks and contribution payments against entitlement. Its aim is to provide an explicit and defined benefit package coverage to those outside formal sector employment, in particular poor and vulnerable population groups. General government revenues transfers are provided to explicitly or implicitly pay full or partial contributions on their behalf. For the beneficiaries, these financing arrangements are thus non-contributory or partially contributory depending on the level of subsidization. As such contribution subsidization breaks or weakens the link between being able to contribute oneself and being entitled to a benefit package [10]. This financing mechanism is in place in more than 40 low- and middle-income countries, and more countries are further exploring or piloting this mechanism [11].

The existing body of literature contains single country studies on health system and health financing reforms [12–15]. Various reviews, e.g. Acharya et al. [16], Tangcharoensathien et al. [8], Bitran [17] assess the impact of diverse health financing reforms on informal sector workers and vulnerable population groups. To fill a gap, this paper specifically looks at health insurance subsidization via government budget transfers as a potential approach to expand UHC with a regional focus on Asia. It explores the patterns, commonalities and differences of such arrangements among countries. The specific focus is on the institutional design of these financing arrangements, i.e. the policy, legal or regulatory specifications that define these arrangements’ structure and the way they operate. The aim is to identify and explore those critical institutional design features that are conductive for UHC progress.

The paper’s focus on Asia combines the South-East Asia and Western-Pacific Regions of the World Health Organization (WHO). Similar regional focus studies using the same methodology have been undertaken for Europe and Latin America [18–20] and are underway for the African Region.

The next section outlines the methods and the analytical framework. The third section assesses the institutional design aspects as well as progress toward UHC. This is followed by a discussion of the possible effects of specific institutional design features and challenges related to progress toward UHC, while the last section concludes with policy lessons.

## Methods

In a first step, all countries from the WHO South East Asia and Western Pacific Regions belonging to the group of low- and middle- income countries, but excluding small island states, were considered if the Global Health Expenditure Database reported social health insurance (SHI) expenditure (termed social security expenditure for health in the Global Health Expenditure Database). In a second step, a comprehensive literature search was undertaken to identify those countries with government subsidization arrangements for vulnerable and otherwise uninsured people. This search rendered the following countries: China, India, Indonesia, Mongolia, Philippines, Thailand and Vietnam. Finally, the remaining countries were screened to see whether there is a country with a government subsidization arrangement, but without SHI expenditure. This last step added Cambodia to the list of countries included in this study.

Search terms such as “health insurance” OR “poor“OR”low-income“OR “subsid*” were combined with the respective country’s name. In another round, the name of the respective scheme was added. This served to collect and collate specific information on the institutional design aspects as per our analytical framework presented in the next sub-section. For the assessment as to UHC progress, we searched and collated data on the indicators described further below. For that matter, the search strategy used different combinations of the following terms: country name OR scheme name, AND “impact” OR “out-of-pocket” OR “utilization” OR “financial protection” OR “catastroph**”. The following databases were screened: PubMed, JSTOR, ISS Web of Knowledge as well as Google Scholar. Thus, the study is based on a literature review and, for the assessment of UHC progress, on a review of secondary databases, using both published and non-published (grey) literature.

The search generated several publications for each country, allowing for triangulation and capturing changes in institutional design as well as in UHC related indicators. Most of the studies are country health financing analyses to review reform experiences, but no studies with explicit impact evaluation design were found here. The analytical framework outlined further below guided the information extraction process from the literature, as well as data compilation and organisation.

To identify plausible contributions and patterns of institutional design features in relation to UHC progress, we plotted the improvements in UHC related progress indicators against the respective institutional design features for the eight countries. Where data points of different years are available, progress towards UHC over time and in relation to changes in institutional design can be assessed. However, it was often difficult to do so due to scarce data availability for most of the indicators. This is a limitation to this study, and this analysis is of explorative nature.

Our analytical framework to assess government subsidization arrangements is broadly guided by the three health financing functions of revenue raising, pooling and purchasing described in Kutzin [21]. As such, it specifically illuminates the following related areas: eligibility, targeting and enrolment rules; financing arrangements; the pooling arrangements; and benefit package design (as one key element of purchasing). The detailed analytical framework is presented in Table 1, which specifies the institutional design aspects and outlines how these potentially relate to progress toward UHC. Progress toward UHC is assessed along the following three indicators: (1) population coverage, understood as affiliation to the health financing arrangement under study here; (2) financial protection; and (3) access to health care services [22].

**Table 1:** Overview of analytical framework

Institutional design aspect	Related policy choices	Intermediate output Indicators	UHC progress indicators
Eligibility and enrolment rules			
Groups eligible for exemption from contributions/subsidization	Definition of vulnerability (e.g. low income, poverty, informal sector, children, pregnant women)	Share of eligible among the bottom two income quintiles and other vulnerable groups	Total population coverage (i.e. enrolment in health insurancefund), differentiated along income quintiles
Targeting method	E.g. universal (based on a very broad criterion such as residence or no employment in the formal sector), indirect (based on socio-demographic, socio-economic or geographic characteristics usually correlated with poverty and vulnerability), direct (through a means assessment or proxy means testing); different targeting approaches can be in place at the same time for different groups	Share of the exempted/subsidized within total (insured) population; share of the exempted/subsidized among those being targeted for exemption/subsidization (targeting effectiveness of the system), Income groups exempted/subsidized	
Enrolment process	Active enrolment by the beneficiary or automatic enrolment by the authorities		
Type of membership of the exempted/subsidized	Voluntary or mandatory		
Organization responsible for identification	E.g. insurance company; central, regional, local government		
Financial arrangements			
Degree of subsidization/co-contribution	Full or partial (a co-contribution is required)	Share of the exempted/subsidized within total (insured) population; share of the exempted/subsidized among those being targeted for exemption/subsidization (importance of budget transfers)	
Type of transfer logic	Individual-based (a specific amount is being paid for each exempted individual) or lump-sum (a lump sum transfer for the entire population is made)		
Calculation logic to determine the amount of funds to be transferred	E.g. based on regular contribution levels, minimum or average wages, specific percentage of the government budget, negotiated by the government		
Financing source of the budget transfers	E.g. general government revenues from central or sub-national levels, earmarked government revenues, transfers from other health insurance funds (cross-subsidization), donor funding	Sufficient funding for a comprehensive benefit package Level of cross-subsidization from contributions	Financial protection (incidence of catastrophic/impoverishing health expenditure) ^a^; Access to services
Pooling arrangements			
Type of pool(s) (general)	Single pool or multiple pools	Degree of fragmentation, Size and composition of pools, Level of cross-subsidization	Equity in access, Equity in financing,
Type of pool (exempted/subsidized)	Exempted/subsidized integrated in the pool with contributors, or separate pool for the exempted/subsidized		
Type of health insurance affiliation membership of the contributors	Voluntary or mandatory		Financial protection
Purchasing arrangements and benefit package design			
Range of services covered by the benefit package	E.g. comprehensive, in-patient focus, out-patient focus, pharmaceuticals, dental care, indirect costs (e.g. transportation)		Financial protection, Access (utilization rates), Equity in access
	Different or same package as that for contributors	Efficiency	
Degree of cost-sharing	Cost-sharing mechanisms (e.g. co-insurance, co-payment, deductible) and rates		
Provider-payment mechanisms	Type of payment and rate		

^a^Catastrophic health expenditure occurs when a household’s total out-of-pocket health payments equal or exceed 40 % of the household’s non-subsistence spending, as per the WHO definition. Impoverishing health expenditure means that out of pocket expenditure shifts a household below the poverty line or even deeper into poverty [23]

The design features and progress indicators are defined and explained in more detail in the next section. It is important to note that subsidized enrolment and coverage in such schemes is only one possible and plausible factor among several to explain improvements in the level of financial protection and access to care of subsidized beneficiaries. The overall economic and fiscal situation is thereby decisive in expanding fiscal space that can be used for state budget transfers to subsidize health insurance contributions.

## Results

### Country overview

In the eight countries included in this study, there are a total of 14 health insurance-type arrangements to which governments provide budget tranfers to extend coverage to people in the informal sector and poor and vulnerable population groups. Most countries are lower-middle income countries, with Cambodia being the only low income country. China, India and Indonesia have several separate subsidization arrangements for different population groups. Table 2 provides an overview of these 14 schemes. All subsidization arrangements are financed publicly and regulated by government. They are also operated by a public agency with the exception of all five Indian schemes that are run by private insurance companies, but are also regulated by government. As such, there is a purchaser-provider split in all countries. Since 2011, India’s Rajiv Aarogyasri is managed by a state agency [24]. Rajiv Aarogyasri was significantly redesigned in 2014 when the Andhra Pradesh State was split into the two states of Telangana and Andhra Pradesh [25]. Since then the scheme is named Aarogyasri Health Care Trust in Telangana [26] and Dr. NTR Vaidya Seva in Andhra Pradesh [27]. This paper assesses Rajiv Aagogyasri’s design features until 2014.

**Table 2:** Country overview

Country (World Bank country income classification) [28]	Name of the scheme(s) (and its abbreviation)	Year of introduction of subsidization arrangements
Cambodia (Low)	Health Equity Funds (HEFs)	2000 [29]
	Government Subsidy Scheme (SUBO)	2006 [30]
China (Upper-middle)	New Rural Cooperative Medical Scheme (NRCMS)	Launched in 2003 (fully implemented in 2008) [31]
	Urban Resident Basic Medical Insurance (URBMI)	Launched in 2007 (fully implemented in 2010) [31]
	Medical Financial Assistance (MFA), which is complementary to NRCMS and URBMI by covering the co-contribution and/or the cost-sharing of the poorest	2003 (rural regions); 2007 (urban regions) [31]
India (Lower-middle)	Rashtriya Swasthya Bima Yojana (nation-wide) (RSBY)	2008 (fully implemented in 2013) [32]
	Yeshasvini Health Insurance in Karnataka State (Yeshasvini)	2003 [24]
	Rajiv Aarogyasri Community Health Insurance in Andhra Pradesh State (Rajiv Aarogyasri) (until 2014)	2007 [32]
	Kalaignar in Tamil Nadu State	2009 [24]
	Vajapayee Arogyasri Scheme in Karnataka State (Vajapayee Arogyasri)	2009 [24]
Indonesia (Lower-middle)	Jaminan Kesehatan Masyarakat (Jamkesmas)	Introduced as Asuuransi Kesehatan Masyarakat Miskin (Askeskin) in 2005; after extension renamed into Jamkesmas in 2007 [33]
Mongolia (Lower-middle)	National Health Insurance Fund	1994 [34]
Philippines (Lower-middle)	Philippine Health Insurance Corporation (PhilHealth)	1996 [35]
Thailand (Upper-middle)	Universal Coverage Scheme (UCS)	2001 [36]
Vietnam (Lower-middle)	Vietnam Social Security (VSS)	Introduced as Health Care fund for the poor in 2002, extended and restructured in 2005 [37]

*The bibliographic references used for each country are indicated in parenthesis in this and the following tables*

Mongolia and the Philippines were the first countries to introduce such arrangements, in 1994 and 1996 respectively. In the first half of the 2000s, Cambodia’s Health Equity Funds (HEFs), Thailand, Vietnam, China’s New Rural Cooperative Medical Scheme (NRCMS), India’s Yeshasvini Health Insurance and the Indonesian scheme followed. India’s remaining four schemes and China’s Urban Resident Basic Medical Insurance (URBMI) were introduced in the second half of the 2000s. China’s URBMI and NRCMS are complemented by the Medical Financial Assistance Scheme, which pays the co-contribution of the poorest and other specific population groups. Likewise, Cambodia’s Government Subsidy Scheme (SUBO), as a way to replicate and expand the number of HEFs, was introduced in 2006. Notably, HEF and SUBO are not yet in place nationwide. As their rationale focuses on paying user fees for health care visits for affiliated poor people, rather than subsidizing contributions for entitlement to a benefit package, they are a border case in this group of subsidized health insurance type schemes.

In Indonesia, there are also local government led subsidization schemes, called Jaminan Kesehatan Daerah (Jamkesda). Yet, these will not be assessed here given lack of data and high variation among these. These local government schemes were introduced in 2004 and directly target the poor and near-poor not covered by Jamkesmas [38]. Major health financing reforms geared towards integrating the country’s various insurance schemes were introduced in early 2014 and are in the process of being implemented. In this paper, we largely focus on Indonesia’s health financing setup prior to 2014 for which information and data is available.

### Eligibility and enrolment

#### Eligible population groups

The eligibility rules are an expression of a country’s perception of which population groups are considered “vulnerable” to be entitled for subsidy, thus determining the number of subsidized people. In general, “vulnerable groups” can be defined as those groups that due to economic, demographic, geographic or other characteristics have an increased susceptibility to not having adequate access to health services with financial protection [39]. However, the subsidized need to be distinguished from the insured family dependents when they are covered via their principal affiliate through the principle of cross-subsidization by contributors. Family dependents usually include children up to a defined age, non-working spouses (most often women), or in some cases elderly parents.

As Table 3 reveals, the most frequent population groups being subsidized are those below the (national) poverty line or the 20 % poorest (five countries), followed by older persons and children (three countries) and those outside formal employment (three schemes). Women form a considerable part of these eligible population groups. A special case is India’s Yeshasvini, where eligibility is restricted to members of rural cooperatives and their family. Vietnam is also unusual with 25 eligible groups defined, including various socio-demographic and socio-economic groups such as children, students, informal sector workers, ethnic minorities in geographically defined areas with high poverty rate as well as veterans, so-called persons of merit and their dependants, state serving individuals and other non-Ministry state serving individuals (e.g. members of the National Assembly and People’s councils) [15].

**Table 3:** Eligibility rules and targeting

Country	Entitled groups	Organisation responsible for identification	Targeting method employed	Type of membership
Cambodia:				
*HEFs*	Poor (under the national poverty line) [40]	Non-Governmental Organizations [29]	Direct: means test (prior—combination of means test screening of a population and consultation with community representatives; or at presentation of treatment—based on asset ownership) [29]	Voluntary [29]
*SUBO*	Poor (under the national poverty line) [40]	National hospitals and health districts [30]	Direct: means test (pre- and post-identification) and supporting letter from authority [30]	Voluntary [41]
China:				
*URBMI*	Urban residents without formal employment, elderly, uninsured young children primary and secondary school students (but not all groups are eligible in all cities), other unemployed urban residents, must be registered in urban area [31]	Local government [42]	Indirect: social and demographic criteria, direct targeting for additional subsidy for the poor [42]	Voluntary [31]
*NRCMS*	Rural population with an agricultural resident registration [31]	Local government [43]	Indirect: geographical (all rural) areas [43]	Voluntary [31]
*MFA (subsidization of co-contribution)*	People living below the poverty line, beneficiaries of social assistance schemes, partly also other groups determined by rural and urban local governments [31]	Local government (township civil affairs office), village committees [44–46]	Mainly direct: economic status assessment Indirect: social, demographic criteria [44–46]	Voluntary [31]
India:				
*RSBY*	Below poverty line families (family head, spouse, 3 dependants) [24]	State governments: responsible for eligible below poverty line household and corresponding data Insurance companies: receive information from state governments Intermediaries: act as social aggregators between insurance companies and beneficiaries [47]	Direct: proxy means test, district below poverty line list prepared by state government and planning commission estimates, using 2002 assessment (perhaps limited below poverty line list, possible target group much larger—more than 75 % of population and more of 93 % of informal workers) [32]	Voluntary [12]
*Yeshasvini*	Rural co-operative society members and families in Karnatka (min. membership of 6-months), below poverty line and above poverty line [24]	Government’s cooperative structure and cooperative societies (guided by a enrolment rate target) [24]	Indirect: cooperative membership as criterion [24]	Voluntary [24]
*Rajiv Aarogyasri*	White ration card holders or annual household income below Rs. 75,000 in urban areas and below Rs. 60,000 in rural areas, entire family, below poverty line and above poverty line [24]	Trust transfers data to insurance company [48]	Direct: means test (existing assessment for white ration card) [12]	Voluntary [12]
*Kalaignar*	Below poverty line or annual household income below Rs. 72,000 and families of 26 welfare boards [24]	State government [24]	Direct: means test [24]	Voluntary [12]
*Vajapayee Arogyasri*	Below poverty line families (up to 5 members) [24]	State government [24]	Direct: means test, registration data from Food, Civil Supplies and Consumer Affairs Department [12]	Voluntary [12]
Indonesia	The poor (2005): national poverty line, near poor (since 2007) [33]	Central government: estimates number of eligible based on district poverty indicators District government: identifies eligible based on welfare and poverty indicators from BPS or family planning board [33]	Direct: proxy means testing (per-capita consumption) plus local government eligibility criteria (there is a quota set for each district based on poverty rates from the national socioeconomic survey) [33]	Voluntary [33]
Mongolia	Citizens covered by social assistance, persons on military service, pensioners, children < 16 years or <18 years if attending general education school, mothers with new-born babies; students Until 2000 also part-time students and herders [49]	Central government [49]	Direct: method unclear (those eligible for social assistance) Indirect: social and demographic criteria [50]	Mandatory [49]
Philippines	The poor (as poorest 25 % of population) and their dependents [51]	Local governments till 2010, now central Department of Social Welfare and Development [35]	Direct: proxy means test (= family income test) [35]	Mandatory [52]
Thailand	Individuals not covered by CSMBS or SSS schemes, registration in primary care network as single requirement [36]	No specific targeting due to universal eligibility [36]		Mandatory [36]
Vietnam	Persons of merit and dependants, veterans, children <6 years (100 % subsidy) [15]	n/a	Indirect: social and demographic criteria [53]	Mandatory [15]
	The poor, ethnic minorities (100 % subsidy) [15]	Local community and district government [15]	Direct: means-test (yearly household economic survey) plus community involvement [15]	Mandatory [15]
	Near-poor (below 130 % poverty line) (70 % since 2012) [54]			Mandatory [15]
	Informal sector workers—agricultural households, members of cooperatives, household enterprises (30 % subsidy) [15]	n/a	n/a	Mandatory [15]
	School children, students (30 % subsidy) [15]	n/a	Indirect: social and demographic criteria [53]	Mandatory [15]

#### Targeting approaches

Most countries employ some form of indirect or direct targeting, and often both, to identify and select individuals eligible for subsidization, but with considerable variation found (see Table 3). Direct targeting is based on (proxy) means testing by determining household income and/or assets) [55]. Indirect targeting is applied to include persons on the basis of socio-demographic (e.g., age, gender), socio-economic (occupation, or employment status) or socio-geographic characteristics. These characteristics are easily observable and identifiable as well as correlated with vulnerability [56].

A few countries employ only indirect targeting, such as India’s Yeshasvini, and China’s NRCMS (with geographic targeting criteria) and URBMI (with socio-economic targeting criteria), whereas their complementary Medical Financial Assistance (MFA) program applies a direct targeting approach. In Mongolia, the main targeting approach is indirect using socio-demographic criteria, but some specific groups are targeted directly. Likewise, the Vietnam Social Security (VSS) scheme applies both indirect and direct targeting for about equal numbers of subsidized people. On the other hand, the Philippines, Indonesia, Cambodia and all Indian schemes (except Yeshasvini) use a direct targeting approach only.

All countries that apply direct targeting use means or proxy-means testing to identify the poor. Several subsidization schemes draw on pre-existing databases built up to grant other forms of social assistance, such as the ration cards system in India [12] or the Equity Access Card, also known as the Poor Card in Cambodia [57]. However, the thresholds that separate eligible and ineligible individuals vary significantly across countries: India’s Rashtriya Swasthya Bima Yojana (RSBY) only includes individuals below the country’s poverty line, whereas Vietnam, Indonesia and the Philippines also attempt to target the near-poor. In these three countries as well as in the complementary MFA in China, the local communities play an important role in the identification process [15, 44, 58, 59].

In a universalist approach, all individuals fulfilling a very broad criterion (e.g. permanent residency in the country, lack of coverage in another SHI scheme) are eligible. Thailand is indeed the only country in this group with no targeting and hence a universalist approach: all permanent legal residents who are not covered by the formal sector health insurance schemes are entitled to the state budget financed Universal Coverage Scheme (UCS). In China, URBMI’s eligibility criteria try to capture all urban legal residents that are not part of the formal sector schemes, thus also coming close to a universalist approach.

### Type of affiliation

Membership in the subsidization programme can be mandatory or voluntary, thus affecting pool size (and the enrolment rate) and its risk composition. Notably, affiliation for contributing members is mandatory in all countries [24, 30, 31, 49, 60–62], except for the individually paying programme in Philippines [63] and non-formal sector workers in Vietnam [15], whereas this varies among the subsidized. The Thai UCS provides mandatory coverage. Likewise, in Vietnam, Philippines and Mongolia, membership in the subsidized arrangements is *de jure* mandatory for eligible individuals without other insurance coverage. However, as a consequence of weak enforcement, it is *de facto* not mandatory. This is particularly the case for the near-poor in Vietnam who are only partially subsidized. In contrast, as per the legal provisions, membership is voluntary in Cambodia, Indonesia, India and China. Yet for administrative reasons and to avoid adverse selection, all household members need to be enrolled in India and China [12, 14]. In China, moreover, local authorities strongly encourage people to enrol which is a result of the incentives given to local authorities to affiliate as many members as possible. As such, it might be perceived as mandatory [64].

### Enrolment process and organization in charge of enrolment

With respect to the enrolment proceedings, two patterns are found, with more details provided in Table 4: 1) active enrolment by the beneficiary him/herself is required; or 2) authorities, in most cases at the local government level, automatically enrol an individual or a household, once identified or recognized to be eligible, without requiring further action by the beneficiaries. The first pattern is usually found in schemes with indirect targeting, namely in Mongolia, Vietnam (for the partially subsidized), China’s URBMI and NRCMS, and India’s Yeshasvini. In the lattercase, the local cooperative secretary would contact and encourage eligible individuals to enrol [65], similar to what is found in China.

**Table 4:** Enrolment procedures

Country and scheme	Type of enrolment	Organization(s) responsible for identification
Cambodia:		
*HEFs*	Automatic enrolment by authorities	Ministry of Planning, village network Trained poverty assessor at health facility (for post-identification)
*SUBO*	Automatic enrolment by authorities [57]	Local government, commune councils, village network (for pre-identification) Trained poverty assessor at health facility (for post-identification) [57]
China:		
*URBMI*	Active enrolment by beneficiary [42]	Local governments [42]
*NRCMS*	Active enrolment by beneficiary [66]	Local governments [66]
*MFA*	Partly automatic enrolment by township government/county leading group of those eligible for social assistance or nominated by village committee (especially for contribution subsidization) Partly active application by beneficiary (especially for additional reimbursement) [44–46]	County civil affairs office, village officer, village committee [44–46]
India:		
*RSBY*	Automatic enrolment by authorities [24]	State government, insurance company, Smart Card Operator, local government [12]
*Yeshasvini*	Active enrolment by beneficiary [48]	Cooperative Societies [65]
*Rajiv Aarogyasri*	Automatic enrolment by authorities [67]	Insurance company [12]
*Kalaignar*	Automatic enrolment by authorities [24]	Insurance company [12]
*Vajapajee Arogyasri*	Automatic enrolment by authorities [24]	Insurance company [12]
Indonesia	Automatic enrolment by authorities [59]	Local governments [59]
Mongolia	Active enrolment by beneficiary [49]	Insurance branch [49]
Philippines	Automatic enrolment by authorities [68]	Local governments [68]
Thailand	Automatic enrolment by authorities [36]	Local primary care network [69]
Vietnam	Active enrolment by beneficiary [15]	Ministry of Labour, Invalids and Social Affairs: those receiving unemployment benefits, social protection or social security allowance Commune people committee: poor, children under 6 etc. [15]

In contrast, the second pattern is found in schemes where direct targeting is employed. In Vietnam (for the fully subsidized), the Philippines and Indonesia, the local government administration actively enrols identified beneficiaries, whereas non-governmental organisations, national hospitals and health districts do so in Cambodia. In the Thai UCS, enrolment is automatic once individuals have registered at a health facility, which they can also do when seeking care [36]. Similarly in Cambodia’s HEFs and SUBO, individuals can be enrolled at the time of seeking care, if they meet the eligibility criteria (post identification).

In India’s RSBY and all Indian state-level schemes, instead, private insurance companies—with the support of local authorities and non-governmental organizations—are responsible for enrolling and providing insurance coverage to eligible beneficiaries [12]. However, this practice has caused a number of problems in RSBY: although private insurance companies registered beneficiaries, they did not immediately deliver the insurance cards that are indispensable to receive benefits. Insurance companies claimed that this delay was due to technical difficulties, yet some authors have suggested that insurance companies benefited by receiving the subsidies for the enrolled whilst not having to provide benefits immediately [47, 70]. However, anecdotal evidence hints to similar challenges in some other countries with public schemes.

### **Financing arrangements**

#### **Level of subsidization**

Another decisive aspect is the actual level of subsidization and the remaining share of co-contribution. In 10 out of the 14 subsidization schemes, the eligible beneficiaries are fully subsidized from government revenues and do not co-contribute (see Table 5). There is an annual registration fee of 30 Rs. in India’s RSBY, which comes to 4–7 % of the amount that the government provides as a subsidy, varying across states [24].

**Table 5:** Financing arrangements

Country	Level of subsidization	Transfer mechanism of subsidy	Calculation logic of subsidy	Financing source of subsidy	Revenue to expenditure ratio (for all subsidized members unless specified, data year)
Cambodia:					
*HEFs*	Full [41]	Individual-based [29]	n/a	Donor and government funds [29]	n/a
*SUBO*	Full	Individual-based [57]	n/a	Central government revenues [57]	n/a
China:			Estimations of future health care expenditure per subsidized member [66] Total annual government subsidy for each subsidized member should not be less than a certain amount, e.g. 120 CNY per year per NRCMS subsidized member (2010) [71]		
*URBMI*	Partial: from 41 % to 85 % in central and western provinces; from 23 to 75 % in eastern provinces (2010) ^*a*^ [72]	Individual-based [72]		Provinces/counties revenues [31]	139 % (average, 2011) [73]
*NRCMS*	Partial: 80 % (2012) [31]	Individual-based [72]		Provinces/counties revenues [31]	123 % (average, 2011) [73]
*MFA*	Covers remaining co-contribution to result in full subsidization [31]	Individual-based [31]		Central and local government revenues [46]	n/a
India:					
*RSBY*	Full, yet small registration fee [24]	Based on the number of families [24]	Insurance companies bidding process [74]	Central government and state revenues [12]	263 % (2009-10)[24]
*Yeshasvini*	Partial: 40 % (2009) [12]	Individual-based [24]	Estimations of future health care expenditure per subsidized member [75]	State revenues [24]	133 % (2009-10) [24]
*Rajiv Aarogyasri*	Full [12]	Based on the number of families [24]	Insurance companies bidding process [24]	State revenues [24]	83 % (2009-10)[24]
*Kalaignar*	Full [12]	Based on the number of families [24]	Insurance companies bidding process [24]	State revenues [24]	102 % (2009) [24]
*Vajpayee Arogyasri*	Full [12]	Based on the number of families [24]	Insurance companies bidding process [24]	State revenues [24]	n/a
Indonesia	Full [61]	Individual-based [33]	Set by government based on expenditures of previous years (6500 Rp. monthly in 2010) [33]	Central government revenues [33]	112 % (2010) [33]
Mongolia	Full [49]	Individual-based	Contribution amount of self-employed is taken as reference (set by government (670 MNT per person per month as of 2012) [13, 34]	Central government revenues [13]	30 % (2009) [13]
Philippines	Full [35]	Individual-based	Based on contributions levels of contributing members [58]	Central government revenues [35]	30 % (2010) [76]
Thailand	Full [108]	Individual-based	Estimations of future health care expenditure per UCS member [77]	Central government revenues [77]	100 % (2010) [77]
Vietnam	Full Partial: from 30 % to 70 % [15, 54]	Individual-based [15]	Based on contributions levels of contributing members (4.5 % of minimum salary) [15]	Central government revenues and social security funds [15]	210 % (poor, 2010) 101 % (near-poor, 2010) 199 % (average, 2010) [15]

^*a*^ *Calculations from authors based on data of indicated reference*

In the remaining four schemes (China’s NRCMS and URBMI, Vietnam, India’s Yeshavini), some or all groups, as listed in Table 5, receive a partial subsidy only and have to partially contribute. In China, the subsidy level is above 80 % in most regions (up to 85 %), but as low as 23 % elsewhere. The complementary MFA pays the remaining co-contribution for a small proportion of beneficiaries (for 6 % of NRCMS and 7 % of URBMI enrolees in 2011) [31]. In Vietnam, the subsidy level ranges from 30 to 70 % of the set contribution depending on the groups, but the poorest individuals as well as other population groups receive a full subsidy. The subsidy level is about 40 % in India’s Yeshavini, whereby the subsidy level varies from year to year depending on available resources and willingness-to-pay findings [75].

#### Type of transfer mechanism and calculation approach

As to the type of transfer mechanism, all countries follow the same logic with the transfer being an implicit or explicit per-capita amount per beneficiary, as per their regulatory or legal provisions. The alternative would be to provide a lump sum that is unrelated to the precise number of number of eligible beneficiaries. There are three approaches found to set this per capita subsidy amount which determines the average amount available for health care expenditure of each subsidized member (see Table 5). One logic is to base the subsidy amount on estimations of future health care expenditure per subsidized member. This method is applied in China as well as in Thailand, although in a more implicit way. In the two Chinese schemes, the level of central government allocations also depends on the regions’ level of economic development as well as affordability considerations [78]. Estimated health care expenditure was also the starting point in Indonesia, and allocations are now adjusted based on expenditure of the previous year. A second logic is to base the subsidy amount upon the contribution rate of contributing members that is applied to a reference salary, such as in the Philippines and Vietnam, or on a set contribution amount (that of non-salaried people), like in Mongolia. Third, in all Indian schemes other than Yeshasvini the actual amount of government revenue transfers is determined through a tender process where insurance companies bid for a “premium” at which they would provide insurance coverage for a family in a pre-defined population group and benefit package [12].

These approaches serve to make government revenue transfers more predictable and needs-adapted by prospectively fixing the per capita subsidy amount, rather than depending on the country’s economic situation or budget allocations and negotiations. However, this has only been partially successful. Independent of the type of approach to set the transfer amount, the actually transferred funds were found to differ from the amount established as per the calculation formula or negotiations. This was the case for example in the Philippines [76, 79, 80], Mongolia [13] and Indonesia [81, 82]. Similarly, in Thailand fiscal constraints resulted in an approved per capita amount that has been lower than the proposed per capita expenditure estimations between 2002 and 2010 [36, 77]. Overall, for the actual subsidy allocation, the regulatory provisions and the related calculation principles appear to have been of limited relevance in some countries, and may be explained by budget constraints, missing data on the number of the eligible or subsidized members, or other political priorities.

#### Financing source of government revenue transfers

In all subsidization arrangements assessed here, the financing source for subsidies are primarily general government revenues. These come from central government revenues in Vietnam, Mongolia, Thailand, Indonesia and the Philippines (since 2011). Sub-national (i.e. state) government revenues are the source used for India’s state-based subsidization schemes. Finally, the remaining schemes—China and India’s RSBY—are financed by both central and sub-national government revenues, whereby the main share comes from provinces/counties and states respectively. A unique feature is found in Cambodia, where subsidies to HEFs are also financed from donor funds. Moreover, in Vietnam, another key source are social security revenues for social security beneficiaries, such as the registered unemployed, pensioners and beneficiaries of social assistance allowances [15]. Notably, the Philippines is the only country which makes use of sin taxes on tobacco and alcohol to subsidize the poorest and most vulnerable groups [35, 83]. After the introduction of this tax in 2012, the government revenues allocated for subsidizing the poor increased significantly [84].

### Pooling arrangements

#### Pooling architecture

Both single fund systems as well as multiple pool systems are present across Asia, with Table 6 providing country details. In three out of the eight countries studied (Vietnam, the Philippines, Mongolia) the subsidized population groups are part of the same pool as the contributors in a single fund system. In contrast thereto, China, Cambodia, Thailand, Indonesia and India have separate, but non-competing schemes for the subsidized population groups, resulting in fragmentation. In these five countries, a formal sector health insurance scheme or non-competing multiple schemes existed already prior to the introduction of the subsidization arrangement. Importantly, however, as of January 2014, the health insurance schemes of Indonesia are being integrated into a single national health insurance agency [85].

**Table 6:** Pooling arrangements

Country and subsidization arrangement	Single/multiple fund(s)	Separate/integrated fund for subsidized	Additional information
Cambodia:	Multiple [30]		
*HEFs*		Separate [86]	n/a
*SUBO*		Separate [57]	n/a
China:	Multiple [31]		
*URBMI*		Separate [78]	Pooling at municipal level, average pool size 2 m [14] Approximately 330 risk pools in 2011 [31]
*NCRMS*		Separate [78]	Pooling at county level, average pool size 500 000 [87] Approximately 2600 risk pools in 2011 [31]
*MFA*			Additional to NRCMS and URBMI membership Pooling at county level [31]
India:	Multiple [24]		
*RSBY*		Separate [24]	n/a
*Yeshasvini*		Separate [24]	n/a
*Rajiv Aarogyasri*		Separate [67]	n/a
*Kalaignar*		Separate [24]	n/a
*Vajapayee Arogyasri*		Separate [24]	n/a
Indonesia	Multiple (single as of 2014) [33]	Separate (integrated as of 2014) [85]	n/a
Mongolia	Single [49]	Integrated [88]	n/a
Philippines	Single [63]	Integrated [35]	n/a
Thailand	Multiple [36]	Separate [36]	n/a
Vietnam	Single [37]	Integrated [37]	Resource allocation and reimbursement regulations result in fragmentation^a^ [37]

^a^Capitation level is linked to historical expenditure, expenditure ceiling is linked to contribution

China’s separate subsidization schemes are further fragmented into sub-national pools. Risk pooling by the NRCMS schemes occurs at county level, with an average pool size of 500,000 individuals, whereas risk pooling by URBMI schemes is at municipal level with an average pool size of 2 million members [14, 89]. There are no risk equalization mechanisms in place across these sub-pools, resulting in unequal per capita average expenditures across counties and municipalities respectively [31].

Among the integrated systems, fragmentation along sub-national lines is equally a concern in Vietnam and the Philippines. Differences in revenue generation capacity that determines resource allocation to provincial sub-pools (in Vietnam) and local governments (in the Philippines), and, in the absence of risk adjusted allocations or risk equalization, result in unequal average per capita expenditure across the country. However, this is the case for all insurance members, not only the subsidized ones.

#### Revenue-expenditure ratio

Revenue sources of the subsidization arrangements include government revenue transfers, co-contributions by beneficiaries where applicable, and in integrated schemes these comprise also contributions from contributors. The last column of Table 5 presents data on the total revenue amount as a share of average expenditure, i.e. the revenue-expenditure ratio, based on aggregate data for all subsidized members. This provides insights into the sustainability of the scheme as well as the level of cross-subsidization between contributors and the subsidized in integrated schemes.

While data, especially recent one, is scarce, this ratio strongly varies across countries and even within countries over time. Some schemes have a balance or a ratio above 100 % and others have a ratio below 100 %. In the Thai UCS, total revenues are more or less equal to expenditure (ratio of 95–105 %), as well as in the Philippines and Indonesia prior to 2009. Interestingly, more frequent is the situation where revenues are higher than expenditure (with a ratio above 105 %), such as China’s NRCMS and URBMI, India’s Yeshasvini and Kalaignar (in 2009), Indonesia (in 2010). This ratio even amounts to around 200 % in Vietnam and above in India’s RSBY. Moreover, in Indonesia, there was a high ratio of 140 % in 2009 when the local government-based Jamkesda scheme came into place, which resulted in double coverage of many people. Another reason for Jamkesmas’ relatively lower expenditure was that not all of the enrolees had a health card and hence had no access to benefits. In 2010 this ratio decreased to 112 %, not because of increased utilization rates, but because the per capita transfer amount was reduced. Finally, some subsidization schemes experience a "deficit": the ratio is below 95 % in the Philippines (2010), India’s Rajiv Aarogyasri (2009-10) as well as Mongolia, which reached only 30 % in 2009.

Ratios above or below 100 % indicate that the revenues (subsidy amounts) and expenditure may need to be better linked, particularly in separate schemes. This is because a persisting deficit endangers the sustainability of the system and results in coverage erosion, under-provision and rationing of benefits by providers. A large surplus could also create efficiency losses since resources could have been used for other purposes.

#### Level and direction of cross-subsidization in integrated pools

Integrated schemes for both the subsidized and the contributors are financed by contributions from contributors as well as by general government revenue transfers, with the possibility of cross-subsidization between different insured groups depending on the level of revenue coming from each source and per capita average expenditure for different groups. Comparing revenue-expenditure ratios for the subsidized with that of the contributors reveals the degree and direction of cross-subsidization (cf. Table 5). In fact, in Mongolia and the Philippines (for 2009 and 2010), this ratio of subsidized members is lower than that of non-subsidized groups, suggesting that the latter cross-subsidized the former. In contrast, the revenue-expenditure ratio of subsidized members in Vietnam was higher than that of contributors, such that cross-subsidization occurred from subsidized members to contributors (in 2009 and 2010). Specifically, formal sector employees, but also the subsidized groups of the poor, children and students (who are only partially subsidized) cross-subsidized pensioners, the voluntarily insured as well as the partially subsidized group of the near-poor (for 2009 only). Moreover, the revenue-expenditure ratio of the poor and children is considerably above 100 % due to low expenditure levels of these groups. As a result, the government budget transfers also indirectly subsidize other groups [15].

### Benefit package design

#### Scope of health services covered

Purchasing consists of the allocation of pooled funds to the health care service providers on behalf of the population for a defined benefit package. The definition of the range of services covered in the benefit package is decisive for financial protection and access to care, as are the cost-sharing mechanisms. The benefit packages are being set by the government in all countries. Moreover, all schemes assessed here provide in-kind-benefits (i.e. health services are directly covered and paid for by the scheme as a third party payer, with no bill settlement of beneficiaries other than paying co-payments). The exceptions are China and the Philippines, where the insured have to advance payments and are later reimbursed by the insurance. Regarding the scope of the defined benefit package (see Table 7), two patterns emerge: The first is a relatively comprehensive package, and the second pattern is a more restricted package primarily covering inpatient care only. The more comprehensive package includes outpatient and inpatient care at all levels and is available in five countries (Vietnam, Mongolia, Indonesia, Philippines and Thailand). These schemes also reimburse pharmaceuticals that are included in the country’s respective essential medicines list. Vietnam’s package is broadest on paper, as all available services are covered in principle, but restrictions come in through a ceiling on the total amount per person per year. The second pattern of a more restricted package is found in all Indian arrangements. China’s NRCMS and URBMI are somewhat in-between the two patterns, with more than half of NRCMS and URBMI counties having some outpatient care in the package [72].

**Table 7:** Benefit package design

Country	Scope of benefit package	
	Services covered	Compared to regularly insured population
Cambodia:		
*HEFs*	District referral hospital medical services, transport costs from health centre to referral hospital, food for patients and carers, sometimes funeral costs [29]	n/a
*SUBO*	Outpatient and inpatient services at health centre level, inpatient only at national hospital, national centres and referral hospital level [30], user fees [40]	n/a
China:		
*URBMI*	Inpatient care and critical outpatient care for accidents or limited chronic/fatal diseases (coronary heart disease, renal haemodialysis) [14] No preventive care More services covered in richer coastal cities [42]	Local governments determine financing level and details of arrangements. Less than UEBMI: no Medical Savings Account, most outpatient services are not covered except for very few selected diseases [14]
*NRCMS*	Inpatient and outpatient services in about 70 % of NRCMS counties, only inpatient services in the other 30 % Listed drugs (approx. 400) [78] There are 4 models of how NRCMS operates throughout China: Model I (in place in 17 % of counties): Inpatient services only Model II (in place in 11 % of counties): Inpatient services and outpatient services for catastrophic diseases (separate deductibles and reimbursement caps) Model III (in place in 7 % of counties): Inpatient services according to a formula, outpatient services and preventive care according to specific formula through collective funds (usually no deductible and reimbursement cap) Model IV (in place in 65 % of counties): Inpatient services according to a formula, outpatient services and preventive care paid through household medical savings account (with deductible and reimbursement cap) [90]	Less than UEMBI [78]
India:		
*RSBY*	Mainly inpatient secondary care: inpatient services on a “day care” basis (subject to sub-limits), transport allowance; pre-existing conditions (minimal exclusions) and maternity covered, care delivered in network hospitals including private hospitals (free choice); recently outpatient consultations [74] Public transport costs up to Rs. 100 per visit and Rs. 1000 per year, post hospitalisation drugs for 5 days [91]	Less: ceiling, no out-patient services, no medicines as in CGHS, no preventive and wellness care and no compensatory cash benefits for loss of wages in case of illness or maternity (ESIS) [24]
*Yeshasvini*	Inpatient secondary and tertiary care: all inpatient charges associated with 823 specified surgical procedures (except transportation) excluded are certain high tech procedures No follow-up investigation; no diagnostic-test for non-surgery-related issues, no medicines Included outpatient consulting at a network of hospitals, since 2007 out-door treatment for stabilization of specific medical emergencies, normal deliveries and paediatric care during first 5-days of live, angioplasty procedure [92]	Less: ceiling, no medicines, no preventive and wellness care (all compared to CGHS) and no compensatory cash benefits for loss of wages in case of illness or maternity (ESIS) [24]
*Rajiv Aarogyasri*	938 hospitalization procedures (surgical and medical), largely tertiary care and some secondary care [24]	Less: ceiling, no out-patient services, no medicines as in CGHS, no maternity, no preventive and wellness care and no compensatory cash benefits for loss of wages in case of illness or maternity (ESIS) [24]
*Kalaignar*	Inpatient tertiary care (626 surgical procedures) [12]	n/a
*Vajapayee Arogyasri*	Inpatient tertiary care including 402 predefined packages and 50 follow-up packages [12]	n/a
Indonesia	Free outpatient primary care in local health centres and third class public hospital inpatient services (registration required) including preventive measures and maternity at public and private (only few) providers [93] No annual physical check-ups, dental prostheses, fertility treatment, indirect costs (e.g. transportation) [60]	Less: Askes and Jamsostek include additional annual physical check-up, under Jamsostek more private providers but larger exclusions of conditions [60] As of 2012, Jamsostek also covers catastrophic cases. [33]
Mongolia	Outpatient services at secondary and tertiary care levels; since 2010 outpatient diagnostic test up to 30 000 MNT per case per month; curative and palliative inpatient care, rehabilitation and long-term care; part of outpatient prescription drug expenses if on National Essential Drug list [88]	n/a
Philippines	Outpatient care, inpatient cute care, emergency care, day surgeries, inpatient care in accredited hospitals [35]	More: outpatient care [35]
Thailand	(Curative and preventive) outpatient and inpatient health services, rehabilitation, certain high-tech medical services (radio- and chemotherapy) but not all, prescription drugs on a national list Beneficiaries must follow the referral system (gatekeeper) to obtain free care Services usually at District Health System (district health centres and hospitals), except for emergencies/accidents [94]	Less than CSMBS More than SSS [36]
Vietnam	All ambulatory and hospital basic, advanced diagnostic curative health services and therapeutic services (including high-tech medical services), referral for higher level services required, drugs inside reimbursement list, transportation costs in case of referral No occupational diseases, medical aid devices, rehabilitation or home care [15]	Same [15]

*Legend: UEBMI Urban Employee Basic Medical Insurance (China), Askes Asuransi Kesehatan (Indonesia), Jamsostek Jaminan Sosial Tenaga Kerja (Indonesia), CSMBS Civil Servant Medical Benefit Scheme (Thailand), SSS Social Security Scheme (Thailand), CGHS Central Government Health Scheme (India), ESIS Employees’ State Insurance Scheme (India)*

Comparison of the benefit entitlements between the subsidized and contributors reveal crucial differences across these two groups in some countries. In fact, benefits are largely the same or similar in two countries: Vietnam and Mongolia. In contrast, the benefit package for the subsidized is more generous in the Philippines (with a larger outpatient benefit package), Indonesia (with more treatment options) and Thailand (compared to the social security scheme) [36, 60, 95]. On the other hand, they are smaller for those of the Thai UCS (when compared to the civil servant scheme) and for the subsidized in India and China, with a much more restrictive coverage of outpatient services. However, outpatient consultations have been introduced in the benefit package of India’s RSBY in 2014 [91].

#### Cost-sharing mechanisms and rates

The cost-sharing mechanisms and rates vary considerably among countries (see Additional file 1). Thailand and Indonesia are the only countries without any formal cost-sharing, and in Mongolia the subsidized members are exempted from nearly all co-payments. In all other schemes, cost-sharing, e.g. in the form of co-insurance and co-payments and benefit ceilings such as in Vietnam, Mongolia and China apply also to the subsidized. The cost-sharing rates range from 5 % of total health insurance expenses for the poor in Vietnam [15] to 50 % in the Philippines [96] and amount to about 50–60 % in both Chinese schemes [14]. But importantly MFA beneficiaries in China receive cash assistance for some part of outpatient and inpatient cost-sharing [31], thus cushioning the financial burden.

In comparison to the contributors, the cost-sharing rates for subsidized members are the same or similar in Thailand and Indonesia. They are higher in China only, but lower in Vietnam, Mongolia and the Philippines. For India, comparative information is not available, however there are no annual benefit ceilings applied in the contributory Central Government Health Scheme (CGHS) in contrast to the subsidized arrangements [12]. Nonetheless, with or without formal cost-sharing mechanisms in place, people also face informal payments in several countries [24, 31, 37, 49] with potentially negative impacts on access, equity as well as impoverishment.

### Progress on universal health coverage related indicators

For the assessment of UHC progress, we collected secondary data on the UHC related indicators. Clearly, the available evidence is limited, and the assessment on progress towards UHC can thus only provide a general idea of the impact trends.

#### Population coverage rates

Here, population coverage is defined as the percentage of the population that is enrolled to a health insurance scheme. Total population enrolment rate vary substantially across countries (see Table 8). They are very high in Mongolia (100 % in 2014), China (99 % in 2012) and Thailand (99 % in 2015). The lowest rates are found in Cambodia (27 % in 2013) and India (25 % in 2014).

**Table 8:** Enrolment rates, uninsured population groups, and exclusion and inclusion errors

Country	Total insured population	Subsidized individuals as share of …			Uninsured population groups	Under-coverage (exclusion error)	Leakage (inclusion error)
		Eligible group	Total population	Total insured population			
Cambodia:	27 % (2013) [41]				1st income quintile women and men (79 % and 86 % in 2010) [97], poor (77 %) [8]		
*HEFs*		76 % (2012) [30]	approx.50 % (2008) [98]	n/a		Approx.25 % [29]	Approx.10 % [29]
*SUBO*		n/a	n/a	n/a		n/a	n/a
China:	99 % (2012) [99]						
*URBMI*		93 % (2010) [72]	16 % (2011) [100]	17 % (2011) [31]	Mainly migrant urban informal sector workers [66]	n/a	n/a
*NRCMS*		95 % (2012) [101]	62 % (2011) [31]	64 % (2011) [31]	n/a	n/a	n/a
*MFA*		100 % (2011) [46]	5–6 % (2011)—those with full subsidization of the contributions [31]	6 % NRCMS and 7 % URBMI enrollees (2011) [31]	n/a	n/a	n/a
India:	25 % (2014) [102]				Formal sector employees (8 %), informal sector [12]		
*RSBY*		28–38 % (depending on poverty estimation model) (2011) [103]	7 % (2010) [12]	27 % (total India) (2010) [12]		49 % of poor 40 % of poorest 10 % (2000) [104, 105]	49 % of non-poor (2000) [104, 105]
*Yeshavini*		25 % (2010) [12]	0.25 % (across all India) (2010) [12] 5 % (Karnataka) (2010) [12]	1 % (total India) (2010) [12]		n/a	n/a
*Rajiv Aarogyasri*		97–100 % (2010) [12]	6 % (India) (2010) [12]	23 % (total India) (2010) [12]		n/a	n/a
*Kalaignar*		n/a	49 % (Tamil Nadu) (2010) [12]	12 % (total India) (2010) [12]		n/a	n/a
*Vajapayee Arogyasri*		n/a	0.12 % (India) (2010) [12]	0.5 % (total India) (2010) [12]		n/a	n/a
Indonesia:	69 % (2013) [106]				Formal sector workers, poor and near-poor (59 % in 2010) [59]	48 % of Q1 (2009) [59]	68 % in Q2-Q5 43 % in Q3-Q5 (2009) [59] 52 % (2010) [33]
Poor		35 % (2010) [59]	15 % (2010) [59]	27 % (2010) [59]			
Near poor			17 % (2010) [59]	31 % (2010) [59]			
All subsidized		33 % (2011) [107]	25 % (2013) [106]	36 % (2013) [106]			
Mongolia	100 % (2014) [108]	n/a	60 % (2014) [108]	60 % (2014) [108]	Self- employed and unemployed, part-time students and herders [49]	n/a	n/a
Philippines	82 % (2015) [83]	140 % (2009) [109]	35 % (2011) [35]	49 % (2011) [35]	n/a	6–30 % (depending on estimation model) (2011) [109]	53–82 % (depending on estimation model) (2011) [109]
Thailand	99 % (2015) [110]	100 % (2008) [111]	74 % (2015) [110]	75 % (2015) [110]	Recently unemployed [111], migrants [112]	n/a	n/a
Vietnam:	76 % (2015) [113]				Near-poor and informal sector just above subsidization income level, elderly below 85 years without pension, disabled people [15]	66 % of Q1 (2006) [59]	41 % in Q2-Q5 18 % in Q3-Q5 (2006) [59]
Poor, ethnic minorities		98 % (2011) [54]	16 % (2010) [15]	27 % (2010) [15]			
Near-poor		25 % (2011) [54]	1 % (2010) [15]	1 % (2010) [15]			
Informal sector workers		33 % (2010) [15]	5 % (2010) [15]	8 % (2010) [15]			
Persons of merit and dependents, veterans, children <6 years		67 % (2010) [37]	13 % (2010) [15]	22 % (2010) [15]			
School children, students		80 % (2011) [54]	11 % (2010) [15]	19 % (2010) [15]			
All subsidized		70 % (2010) [15]	41 % (2010) [15]	70 % (2010) [15]			

Another dimension is to capture the share of the actually subsidized insured members as compared to those who are theoretically entitled or targeted as per entitlement. This gives insights into the targeting effectiveness. The Thai UCS with a universalist approach is the only one with a 0 % exclusion error [77]. In schemes with indirect targeting, the coverage rates of the effectively subsidized as a share of the theoretically eligible number of people vary: it is high for China’s URBMI and NRCMS, covering 93 and 95 % respectively as well as for school children and students in Vietnam (80 %). In contrast, India’s Yeshasvini reaches only 25 % of the intended target group.

Among subsidization schemes with direct targeting, India’s Rajiv Aarogyasri, Vietnam for the poor and China’s complementary MFA reach 100 % or close to 100 % of their target number. Coverage of target groups is much lower in India’s RSBY, but also in Cambodia’s HEFs, which is due to the fact that there is no nation-wide coverage of HEFs nor SUBO. For the group of the partially subsidized near-poor in Vietnam, the coverage rate is only 25 % in 2011. On the other hand, having a closer look on targeting effectiveness also reveals that most schemes have suffered from severe inclusion errors in the past (see Table 8).

Another population coverage indicator is the share of the subsidized as of the total population to reveal the magnitude of the subsidization arrangement. In most countries, this share ranges between 15 and 50 %. This ratio is higher in Thailand and China’s rural population, 74 and 62 % respectively, but extremely low in India’s Vajapayee Arogyasri Scheme (0.12 %) and Yeshasvini (0.25 %). Thus, in all countries with the exception of these two Indian schemes an important share of the population relies on subsidization arrangements to obtain health insurance coverage.

It is equally important to keep in mind the share of the remaining uninsured (see Table 8) and reasons thereto. This share is tiny (about 1 %) in Thailand given its universalist approach, whereby migrants and those who recently became unemployed and transition to the UCS belong to the uninsured. In the Chinese schemes, largely relying on indirect targeting, the requirement of residency status makes it difficult for internal migrants to enrol (about 1 %). Coverage rates in Mongolia reduced in the early 2000s, as herders and part-time students were no longer subsidized and had remained uninsured during these years, and the self-employed and unemployed were also among the uninsured [49, 114].

As Table 8 reveals, the highest rate of uninsured people is found in countries with direct targeting: 62 % in the Philippines [115], 31 % in Indonesia, up to 75 % in India [12] and 73 % in Cambodia. In Vietnam, where direct and indirect targeting each account for about half of subsidized members, the share of the uninsured (33 %) is higher than in schemes with indirect targeting, but lower than in schemes with direct targeting [15].

#### Financial protection

Global evidence has shown that there is a strong correlation between out-of-pocket (OOP) expenditure as a share of total health expenditure and the share of households experiencing financial hardship [23]. Thus, a starting point is to look at trends in OOP expenditure as a share of total health expenditure. Table 9 presents data on changes over time in OOP expenditure and financial protection measures of the total population, the subsidized target groups or of poor population groups. However, the introduction of subsidized coverage may be just one factor among several to explain changes in the level of financial protection.

**Table 9:** Financial protection

Country and arrangement	Change in OOP spending since the year where the subsidization arrangement was introduced	Incidence of catastrophic expenditure (at a 40 % threshold, unless otherwise stated)	Incidence of impoverishing expenditure
Cambodia:			
*HEFs*	Reduced by 35 % on average Reduced by 42 % for poorer households [29]	n/a	Reduced (no year indicated) [116]
*SUBO*	Reduced by 18 % for the poor [117]	n/a	n/a
China:			
*URBMI*	n/a	n/a	n/a
*NRCMS*	Mixed evidence: Similar [118], Not reduced [90] Decreased cost of a delivery, increased cost of an outpatient visit (more expensive type of care) OOP effects smaller for 1st income quintile [119]	2006: no reduction at 10 and 20 % thresholds [120]	n/a
India:		Protection from catastrophic spending is limited since in India the main determinants of catastrophic spending are outpatient services and medicines which are not covered by the vast majority of the schemes [24]	2004: 5 % of total population pushed below the poverty line [24]
*RSBY*	Total and outpatient expenditure decreased slightly stronger in RSBY districts versus non-RSBY district (but maybe subject to confounding effects) [121]	n/a	n/a
*Yeshasvini*	n/a	Lower borrowings/payments out of savings in case of surgery [65]	n/a
*Rajiv Aarogyasri*	Small reduction	n/a	n/a
*Kalaignar*	n/a	n/a	n/a
*Vajapayee Arogyasri*	n/a	n/a	n/a
Indonesia	n/a	Declined (and low compared to average OOP) [60] Lower among Jamkesmas beneficiaries than for other insured groups [33]	n/a
Mongolia	2012: OOP payments in rural areas slightly smaller than in urban areas and ten times higher in 5th income quintile than in 1st income quintile [122]	2009: Five times higher in 1st than in 5th income quintile, two times higher in 1st income quintile than across all quintiles [49] 2011:1.6 % of households in 1st quintile [88] 2012: 5.5 % of total households (at 10 % threshold); 1.1 % of total households (at 40 % threshold) [122]	2012: approx. 1 % [122]
Philippines	n/a	2009: 1st income quintile: 0.5 %; 5th income quintile: 2 % [35]	n/a
Thailand	2000–2004: OOP share of total or non-food household consumption decreases significantly, especially in the 1st and 2nd income quintiles (30 % reduction) [123] 2000–2006: 1st quintile reduction of about 3/4 ^*a*^ [124]	Incidence and intensity of catastrophic expenditure declines particularly among 1st and 2nd income quintiles especially in rural areas [123] Dropped from 6.8 % in 1996 to 2.8 % in 2008 among UCS members in the poorest quintile (at 10 % threshold) [125]	2004–2009: decreasing in households with one or more UCS member(s) [125]
Vietnam	Similar average OOP spending for all quintiles [53]	From 2002 to 2010: hardly changed in 1st and 2nd income quintiles [37] 2010: 1st quintile 4.7 %; 2nd quintile 4.5 % [54]	From 2002 to 2010:% of households has hardly changed for the 1st income quintile and decreased from 11 % to 6 % for the 2nd quintile [37] 2010: 1st quintile 5.4 %; 2nd quintile 6 % [54]

^*a*^ *The remaining catastrophic health expenditure is mainly due to accessing designated services without proper referral (use of private services or public services outside province) and services not covered by benefit package. There is a need to increase quality of public institution and confidence in their services and extend benefit package*

A clear decrease in OOP, comparing average values in the years before and after the introduction of the subsidization scheme particularly for the poor, was found for Thailand and in Cambodia’s HEFs and SUBO. For beneficiaries of India’s Yeshasvini and Indonesia’s Jamkesmas, there was some reduction. In Indonesia, it was found that some beneficiaries preferred not to use their cards and instead paid out-of-pocket. They wanted to avoid perceived stigmatization from health providers and longer waiting times as a result of having to complete additional administrative forms [33].

Only a very small OOP expenditure reduction was observed for the beneficiaries of India’s Rajiv Aarogyasri and RSBY and for China’s URBMI and NRCMS. In the Philippines no change or even a small increase was reported for the population, but when differentiated along deciles, the poorest 10 % and richest 40 % of all Philippine Health Insurance Corporation (PhilHealth) members (regardless of subsidization status) experienced a reduction of OOP payments. In contrast to this, in Vietnam, between 2002 and 2010, OOP expenditure increased across all income quintiles [126].

However, overall, in most of the subsidization schemes, beneficiaries still incur considerable OOP expenditure, even though cost-sharing rates are lower for the subsidized than for contributors. This remaining level of cost-sharing might be particularly problematic for the subsidized low-income groups, since they are the ones that most likely forego care [127].

As to the financial protection measures, namely catastrophic and impoverishing health expenditure (for the definition see Legend of Table 1), the evidence is scarce and mixed. Regarding, the incidence of catastrophic expenditure, a clear-cut reduction was only observed for UCS beneficiaries in Thailand, the most notable effect being reported among the poorest beneficiaries again. Some small reductions could also be shown for China’s URBMI beneficiaries, as well as in Vietnam and Indonesia. However in Indonesia, this was partly attributable to lower utilization rates. For beneficiaries of India’s RSBY and Rajiv Aarogyasri, no change in the incidence of catastrophic expenditure was found. In China, NRCMS beneficiaries in the Western regions [120] and those in income deciles 3–10 experienced an increase [118, 120], while another study reports a decrease for the poorest 10 % [118]. In sum, financial protection improved somewhat in some countries and for the poorest, but in a few others, it did not.

#### Utilization rates

It is more difficult to measure effective access to needed services in the absence of data on actual needs. A proxy for access to services are utilization rates [128]. Utilization rates of the subsidized for outpatient and/or inpatient care increased since the introduction of the subsidization schemes and were higher compared to the uninsured in six schemes, namely in Vietnam, Indonesia, China, and Thailand as well as India’s Yeshasvini and Cambodia’s HEFs. In Thailand and China’s NRCMS, the poor have experienced the highest increase in utilization. For China’s NRCMS, Vietnam and Indonesia some studies also report there was no impact at all in certain regions and that among poorer beneficiaries the increase was much more moderate (Table 10). Likewise, in Mongolia, utilization rates for family group practice and district hospitals became more pro-poor, whereas for tertiary care, they became even more pro-rich during the period of 2007–2012 [129].

**Table 10:** Utilization of health services

Country and arrangement	Utilization of health services (with a focus on curative outpatient and inpatient care)
Cambodia:	
*HEFs*	Increased utilization rates for the poor [86]
*SUBO*	No increased utilization rates for the poor [57]
China:	
*URBMI*	China Health and Nutrition Surveys of 2006 and 2009: significant increase of utilization of outpatient and inpatient care especially for children, members of low-income families and residents in the relatively poor western region [100]
*NRCMS*	Inconclusive evidence: Increased use of preventive care (mainly physical check-ups) No increased utilization of formal care [90] Increased utilization of inpatient and outpatient services For the poor greater utilization in outpatient services mainly concentrated on lower level (village/township) facilities [118]
India:	
*RSBY*	2.5 hospitalisations per 100 beneficiaries Strong variations among different states (overall average for India: 2.3 for rural and 3.0 for urban regions) [121]
*Yeshasvini*	2003–2009: utilisation rate increased [65] 2.2 hospitalisations per 100 beneficiaries Surgeries per 100 insured: 2003/4: 0.5; 2005/6: 1.2; 2007/8: 2.4 Outpatient visits per 100 insured: 2003/4: 2; 2005/6: 3.5; 2007/8: 8.62
*Rajiv Aarogyasri*	0.5 hospitalisations per 100 beneficiaries
*Kalaignar*	0.4 hospitalisations per 100 beneficiaries 45 % of claims are being made by women
*Vajapayee Arogyasri*	0.4 hospitalisations per 100 beneficiaries [12] No significant increase in use of covered health services [130]
Indonesia	Increase for rural public health centres and urban public hospitals [61] Only an estimated 60 % of beneficiaries used health services using their health insurance card in 2006 [93] Slightly higher outpatient utilization rates of 16 % among Jamkesmas beneficiaries compared to contributors in formal sector scheme (14 %), local government managed Jamsostek scheme (14 %) and the non-insured (12 %) in 2010 Inpatient utilization rate of Jamkesmas beneficiaries are lower than that of Askes and Jamsostek enrolees—2.7 % compared to 4 and 3.3 % respectively, but higher than that of the uninsured (2 %) in 2010 [33]
Mongolia	Larger utilization among elderly, low-income and vulnerable groups Low utilization among self-employed and students Health service utilization quite similar across quintiles [49]
Philippines	Evidence for underutilization among subsidized [131] 2003 survey found 35 % of 5th income quintile and only 17 % of 1st income quintile used health services in the last 12 months For the first quarter of 2009, 58 % of claims were filed by employed members, 18 % by individually paying members and 14 % by subsidized members, while membership shares were 48; 22 and 19 % respectively [63] The hospital admissions rate among poor households is less than half that of the total population, and three times less than for the richest 10 % (for 2007) [35]
Thailand	Significant increase, especially in district health-care system; pro-poor distribution of service utilization [124] Significant increase in utilization of outpatient and inpatient services after introduction of the UCS, particularly at primary care units and district hospitals [77] Increased health care utilization especially among the previously uninsured Overall outpatient and inpatient services among UCS members rose steadily from 2003 to 2011, with the outpatient utilization rate increasing from 2.45 to 3.23, and inpatient admission rate increasing from 9.42 to 11.40 % 2003–2009: significantly higher outpatient and inpatient utilization rates among the poorest quintile compared to richest quintile [36] Still differences in average utilization rates across the three schemes: Outpatient care utilisation rates in 2011: UCS—3.12; SSS—2.68: CSMBS—4.91 Inpatient utilization rates in 2011: UCS—0.11: SSS—0.05; CSMBS—0.16 [132]
Vietnam	Inconclusive evidence: Increase in utilization, stronger for inpatient than for outpatient care; stronger for better-off, almost insignificant for poorest decile [53] No change in utilization [133] Lower utilization rates among poor and near-poor than the average utilization rates of all insured people [54]

*Legend: CSMBS Civil Servant Medical Benefit Scheme (Thailand), SSS Social Security Scheme (Thailand)*

A comparison of utilization rates between subsidized beneficiaries and contributors reveals some inequities in some countries. In fact, utilization rates are still lower for the subsidized members in Thailand, Vietnam, Indonesia (with regards to inpatient care) and considerably lower in the Philippines. In China, differences in the use of outpatient and maternity care services between different regions and lower and higher income groups have notably reduced since NRCMS and URBMI were established [101]. However, the limited scope of the NRCMS benefit package limits improvements in access to outpatient services.

## Discussion

This section aims to explore the contribution, and in view of scarce data, the plausible effects of critical institutional design features of such financing arrangements on progress towards UHC. Importantly, there are many other factors, relating to the supply side, service perception, and other developments in the health sector and beyond, which can affect and explain UHC progress.

### The role of the targeting approach on population coverage

In schemes with indirect targeting approaches, identification criteria are usually easily observable characteristics. Thus it is rather the enrolment process than the actual targeting process that appears decisive for exclusion errors, i.e. gaps in covering the envisaged target group. This will be further elaborated below. An exception to this might be Vietnam where the existence of 25 membership categories can make it difficult to decide through which membership category to enrol [15].

In contrast, correct targeting of beneficiaries in schemes using direct targeting appears more challenging and targeting effectiveness is weaker. In just about half of the schemes only little more than 50 % of the intended target group is covered, with considerable inclusion and exclusion errors. Detailed data is available from India and Indonesia and reveals that targeting effectiveness depends on a number of factors, such as the reliability and validity of the (proxy) means-test instrument [59, 104, 105, 134], availability and accuracy of updated data [135] as well as administrative capacity to undertake the identification and enrolment process. In the past, lack of clear procedures had been reported as a core challenge for Indonesia and the Philippines for example [60, 68, 136]. To address these concerns, the Philippines has developed a centralized targeting system in 2011. This system is under the responsibility of a ministry and every individual can apply to be screened [35, 63, 95]. In Indonesia’s Jamkesmas, inclusion and exclusion errors are have been caused by variations and discretion in the proxy-means-testing criteria and poor knowledge among the targeted beneficiaries [33]. This points to the importance of standardized procedures, rigorous administrative control as well as information provision to the public to ensure transparency and to avoid vulnerability to ad-hoc decisions.

Nonetheless, the actual level of wrong exclusion and inclusion might be less severe than at first sight. This is because income distributions are often “clustered” around the poverty line [104], i.e. differences in the income situation between households just below and just above the threshold might be small, such that there is no actual miss-targeting occurring.

In summary, the technical and administrative challenges as well as related costs of direct targeting, identification and monitoring render efficient coverage of the target groups much more difficult than a universalist approach or indirect targeting. Due to the considerable share of wrong exclusion among the poor, countries are in the process of modifying and improving their targeting process, for example by sharpening proxy-means test procedures.

### The role of enrolment procedures on population coverage

Country evidence suggests that enrolment procedures appear to be decisive for enrolment rates. For example, in China’s NRCMS and Mongolia, population coverage rates increased also as a consequence of simplified enrolment procedures [87]. In Mongolia, capitation payment to family group practices indirectly incentivised them to reach out to their catchment population to enrol them, however this stopped when payment changed to budget allocations. It is difficult to assess the effect of “automatic” enrolment by the administrative authorities compared to active enrolment required by beneficiaries. Certainly, however, active enrolment by the eligible beneficiary him/herself requires the individual to be informed about one’s eligibility status and to have the capacity to follow the enrolment procedures. On the other hand, “automatic” enrolment by the authorities does not imply that beneficiaries are aware of their insurance/coverage status and benefit package entitlement. Hence, some of them may not use services. Another challenge is that entitled beneficiaries may be affiliated, but do not yet have the health insurance card to access services, which has been a concern in some schemes in India [12]. Moreover, the delivery of health cards has been reported to be prone not only to delays and technical problems [15, 59], but also to bribes [47]. Finally, when enrolment of the poor and near-poor is voluntary, adverse selection results, as in Indonesia, and it was found that some people only enrol when they need health services [33].

However, when local authorities benefit from higher enrolment rates, e.g. by receiving higher central government revenue transfers, they have an incentive to actively encourage and foster enrolment, particularly when enrolment is voluntary. In principle, the organization that enrols beneficiaries should have a positive incentive or no disincentive to enrol members and encourage eligible individuals to sign up. This is usually the case when the organization responsible for enrolment is not in charge of financing budget transfers or when it receives additional funds in form of matching grants or commissions. Moreover, enrolment targets or reputation and recognition related to enrolment levels also constitute incentives. For example, central government budget transfers were conditional upon achieving a target enrolment rate in China’s NRCMS [137, 138], and the local governments were reported to have very actively encouraged households to enrol, with membership becoming mandatory in practice [139]. Similarly, in India’s Yeshasvini, cooperative secretaries had to achieve enrolment targets and consequently put efforts into convincing cooperative members to enrol [75]. In contrast to this, in the Philippines prior to 2011, local governments were responsible for both enrolling the poor into PhilHealth as well as financing their subsidies. It was found that without central government monitoring, a large share of the poor were not enrolled [63]. Enrolment rates only improved when the central government ran enrolment campaigns, whilst at the same time financing most part of the subsidy [47, 68, 70, 95]. Since 2011, budget transfers are again completely financed from central government revenues.

### The role of the subsidization level on population coverage

Another decisive aspect is the actual level of subsidization and the remaining share of co-contribution. Since individuals or households may not be able to afford or may not be willing to pay even parts of the contribution amount, the assumption is that partial subsidization leads to relatively lower enrolment rates.

But country evidence also shows that schemes with partial subsidization can reach high enrolment levels due to other factors coming into play. Partial subsidization is applicable in four schemes only: China’s URBMI and NRCMS, India’s Yeshasvini and in Vietnam for the near-poor, informal sector workers, school children and students. Given the high enrolment rates in China and of school children in Vietnam (above 90 % and 80 % respectively), affordability may not have been a key barrier [37]. Instead, enrolment practices and encouragement, which have been pointed out further above for China, could be an important factor. Similarly, it is reported that pupils in Vietnam can be easily addressed and encouraged to enrol [15]. Moreover, the actual level of subsidization could be critical. Subsidization levels are very high in the majority of councils of China’s URBMI and NRCMS (above 80 % of the total “contribution” amount per capita), against only 40 % in India’s Yeshasvini. On the other hand, a randomized control study from Vietnam showed that an increase in the level of the current subsidization level, i.e. 70 % for the near poor, together with better information on enrolment procedures and benefits did not have a significant effect on enrolment rates [140]. There are hence other decisive determinants for enrolment of informal sector workers in Vietnam as elsewhere, when this is voluntary and only partially subsidized. People in the informal sector cannot be as easily reached as school children. Likewise, access to care, quality of care provided and the attractiveness of the benefit package and the offered financial protection are equally relevant [17].

Notably, in Mongolia, herders and part-time students were eligible for subsidization in the beginning, but later on no longer because it was considered that their capacity to pay for health insurance contributions on their own increased as a result of privatized animal stocks and expanded employment opportunities. This path is an interesting alternative by starting with fully subsidizing health insurance contributions for specific population categories, before then gradually narrowiding down the subsidized group in line with improvements in household income and people’s living standard. However, this approach also faces the challenge to maintain high population coverage attained as a result of the earlier broad subsidization policy.

### The role of the pooling architecture on access

Having established integrated pools for both the subsidized and contributing members, countries avoided fragmentation and enhanced risk pooling and redistributive capacity. This is the case for three countries (Vietnam, Philippines, Mongolia), which operate a large single pool with a more balanced risk composition. Integrated schemes also allow for cross-subsidization between contributors and subsidized members. However, the findings also revealed that cross-subsidization may occur from one subsidized population groups to other (subsidized or contributing) population groups.

In integrated schemes, notably, the benefit package for subsidized member is at least equal to that of contributing members. In contrast, a separate scheme for subsidized members bears the risk of designing a benefit package that is smaller than that of the contributory scheme, thus easily establishing or further institutionalising a segmented health system resulting in unequal access to health services [66, 141]. This is the case for China and India. But this is by no means automatic. In fact, Thailand and Indonesia have set up separate schemes, but benefit packages are similar. These examples show that political will is also highly relevant to successfully ensure that a similar benefit package is offered even when schemes are separate [62, 142].

### The role of the purchasing structure on access and efficiency

A further difference between integrated and separate schemes is the resulting market structure of purchasing, i.e. the number of purchasing agencies and the degree of competition between them. The integrated schemes are all operated by one insurance scheme, i.e. a single payer system. In contrast, in the countries that introduced separate schemes, the system was already fragmented, such that the creation and introduction of an additional separate scheme increased this fragmentation. As a result, multiple purchasing agencies exist and often employ different provider payment mechanisms. Yet these multiple purchasers are non-competing, since people are eligible for only one of the schemes. Thus, the assumingly most important advantage of a multiple purchaser system does not apply here. Instead, compared to single payer systems, this might result in a weaker purchasing power to promote efficiency and high quality of services. Two other draw-backs of multiple payer systems relate to reduced economies of scale and higher administration costs [21]. However, for the very large separate schemes such as in China, Indonesia, Thailand and India (RSBY, Rajiv Aarogyasri, Kalaignar), these are likely to be less relevant for the subsidized schemes.

One core concern about a multiple fund/purchaser system relates to setting coherent incentives for providers, when purchasers apply different provider payment mechanisms and remuneration rates. This implies that patients from one insurance scheme may be more “attractive” to providers than patients from other schemes. An example of this distorting effect was found in India, where hospitals contracted by RSBY have been reported to turn down RSBY patients after a certain self-defined number of patients was exceeded. This is because the payment received for RSBY patients was notably lower than that obtained for individuals with a different insurance or patients paying out of pocket [70]. Another example is Cambodia’s SUBO, which is reported to have weak administrative procedures, resulting in delays of reimbursing providers. These show as a result little commitment to provide services to subsidized beneficiaries [143].

However, in one respect, country evidence suggests that the creation of a new and separate scheme may have had an advantage. Some of the separate schemes were able to move away from engrained but less efficient provider payment methods. In Thailand, it was the UCS that spearheaded provider payment reforms as well as the introduction of a DRG-based payment system, which was only later applied in the civil service scheme [69]. The difference is notable: Annual per capita expenditure of the civil servants scheme was three times higher than that of UCS in 2006. Similarly, Jamkesmas has been the first scheme to introduce DRG in Indonesia [144].

### The role of benefit package design on financial protection and access

The schemes that were found to effectively reduce the incidence of catastrophic expenditure while at the same time increasing utilization rates offer a comprehensive benefit package including both inpatient and outpatient services. This is the case for Thailand, Indonesia, India’s Yeshasvini as well as Vietnam. The absence of or the low level of cost-sharing in Thailand, Indonesia and Vietnam for the subsidized or for the poorest also appears to play a role in improving financial protection, although for Indonesia the evidence is somewhat inconclusive. Evidence also shows that utilization rates for the poor have increased in these three countries. However, in Indonesia, utilization rates of the subsidized are still low due to barriers in geographical access, lack of trust in health services as well as lack of information on benefit package. Other studies also found that lower cost-sharing levels reduce service under-utilization since co-payments often deter in particular the poor from accessing health care [145, 146].

In contrast to this, India’s RSBY and China’s NRCMS benefit packages focus on inpatient care and have been less successful in providing financial protection. Expenditure on outpatient care and medicines account for the largest share in total OOP [43, 147]. Likewise, in these two schemes, cost-sharing levels are higher and benefit ceilings lower than those for the regularly insured population, assumingly a reason for no or very small improvements in financial protection.

Health care benefits in-kind, i.e. the coverage of health service provision without billing (other than co-payments), in contrast to payments at the point of use and retrospective reimbursement thereafter by the insurer, constitutes another important design feature that is conducive to increasing financial protection and/or service utilization by the poor. In fact, incidence of catastrophic expenditure did not reduce in the two countries with schemes in which beneficiaries have to file insurance claims, namely China’s NRCMS and PhilHealth. One explanation relates to low education status, as it compromises the successful filing of claims as found for the Philippines [131]. There is also evidence that subsidized PhilHealth members under-utilise health services [148] given the inability to advance payments in low-income groups [131]. For China’s NRCMS, there is evidence that immediate instead of delayed reimbursement increases the likelihood of seeking outpatient care for the second quintile, but not for the poorest quintile. This is due to the degree of cost-sharing [149].

## Conclusion

Subsidization of health insurance type arrangements through state budget transfers for people in the informal sector and for vulnerable population groups has become a more frequent financing mechanism in Asia, particularly over the past 10 years, with 8 countries and 14 arrangements in this region. Moreover, three other countries, namely Bangladesh, Laos PDR and Nepal are exploring or preparing the introduction of subsidization arrangements [150–152]. This paper has shown that this financing arrangement is a mechanism to expand coverage through a non-contributory approach or through the combination of contributory and non-contributory approach. Budget transfers from central government revenues allow the central government to provide more subsidies to those geographical regions with limited economic potentials compared to economically developed areas.

One of the key institutional design features distinguishing the various arrangements is the targeting method, with a universalist approach reaching the highest total and group-specific enrolment rates. Financial constraints and the large number of individuals that need to be covered still seem to drive the decision about the targeting approach and the income threshold. A continuous challenge related thereto are the high poverty rates prevailing in these countries. Hence, in view of the current eligibility criteria relating to poverty threshold levels, countries solely relying on a direct targeting approach may continue not covering a considerable share of the population even in the medium to long run, and their goal of universal health coverage may be difficult to reach [8].

Yet, there are also concerns around indirect targeting. For example, despite its strong acceptance and support among the population [55], categorical targeting of children until the age of 18 (other than under-fives) may be questionable from a distributional point of view, as households with children from upper income quintiles will equally benefit from these subsidies, thus undermining any attempts to shift to pro-poor spending. One possible approach to improve equity and targeting, for example in countries like Mongolia and Viet Nam, might be the gradual shift from individual membership to family membership, such that dependents of better-off families would be cross-subsidized through family insurance and no longer be covered through state budget transfers.

Another concern is that the budget available for subsidization of vulnerable or low-income groups is often constrained, whether due to the general economic situation, weak tax-raising ability or political priorities. Governments often face a trade-off between the number of subsidized individuals versus the financial resources available per subsidized member. Nonetheless, a targeted scheme may represent a point of departure and gradual extension of eligibility may ultimately lead to a subsidized scheme based on a universalist approach. The experience of Thailand shows that this is a path towards UHC, and other countries seem to embark on this path as well, with Indonesia, Vietnam and the Philippines having substantially increased and actually doubled the number of subsidized members over the past 10 years. This path will require constant political commitment and further resource mobilization.

In view of the smaller benefit entitlements for subsidized members in separate schemes compared to those for non-subsidized members, integrated schemes are the more preferable option. Again, political realities may render an integrated pool for both formal sector contributors and subsidized members more difficult, particularly in countries that have already separate schemes. Establishing a separate fund is certainly preferable to not at all expanding coverage to those outside the formal sector. But it is then important to work towards harmonization of the benefit packages in order to prevent inequitable access to health services.

Finally, this assessment showed that subsidization arrangements can contribute to improved financial protection and utilization for the subsidized and thus reduce inequities in access to health care services (i.e. inpatient and/or outpatient care) across different income groups if well designed and implemented. But further improvements in the institutional design features of these schemes are needed, particularly with respect to their benefit package and co-payment rules. Ultimately, in order to better assess the impact of these arrangements, more systematic evaluations and more evidence are needed. Future research could also assess the governance of the whole health financing system as well as of the particular scheme to generate evidence how this influences progress towards UHC.

## Additional file

Additional file 1: Degree of cost-sharing. Provides information regarding the cost-sharing mechanism and rates in each country [153–155]. (PDF 31 kb)

## Abbreviations

Askes: Asuransi Kesehatan (scheme for civil servants in Indonesia)
CNY: Chinese yuan renminbi (currency)
CGHS: Central Government Health Scheme (scheme for central government employees in India)
CSMBS: Civil Servant Medical Benefit Scheme (scheme for civil servants in Thailand)
DRG: Diagnosis related group
ESIS: Employees’ State Insurance Scheme (India)
HEFs: Health Equity Funds (Cambodia)
ILO: International Labour Organization
Jamkesmas: Jaminan Kesehatan Masyarakat (Indonesia)
Jamsostek: Jaminan Sosial Tenaga Kerja (scheme for formal sector workers in Indonesia)
MFA: Medical Financial Assistance (China)
MNT: Mongolian tugrik (currency)
NRCMS: New Rural Cooperative Medical Scheme (China)
OOP: Out-of-pocket expenditure
PhilHealth: Philippine Health Insurance Corporation
Rp.: Indonesian rupiah (currency)
Rs.: Indian rupee (currency)
RSBY: Rashtriya Swasthya Bima Yoana (India)
SHI: Social health insurance
SSS: Social Security Scheme (scheme for formal sector workers in Thailand)
SUBO: Government Subsidy Scheme (Cambodia)
UCS: Universal Coverage Scheme (Thailand)
UEBMI: Urban Employee Basic Medical Insurance (China)
UHC: Universal health coverage
URBMI: Urban Resident Basic Medical Insurance (China)
US$: United States dollar (currency)
VND: Vietnamese dong (currency)
WHO: World Health Organization
