# Supplementary material for: Subsidized health insurance coverage of people in the informal sector and vulnerable population groups: trends in institutional design in Asia
Source: Int J Equity Health. 2016 Oct 4;15:165. doi: 10.1186/s12939-016-0436-3 (PMC5050723; doi:10.1186/s12939-016-0436-3)
Supplement: Additional file 1: — Degree of cost-sharing. Provides information regarding the cost-sharing mechanism and rates in each country [153–155]. (PDF 31 kb) [file 12939_2016_436_MOESM1_ESM.pdf]

Additional File 1. Degree of cost-sharing

Country and arrangement	Cost-sharing	
	Co-payments, co-insurance, deductibles, ceilings	Same/ different to regularly insured population
Cambodia:		
-HEFs	No user fees (30)	n/a
-SUBO	No user fees (40)<br>Flat rate per case (considered too low) (57)	n/a
China:		
-URBMI	50%-55% average cost-sharing of total health bill for inpatient costs (deductibles, coinsurance provisions, reimbursement caps; heterogeneous across regions); increases at higher care levels (42)	Higher: in UEBMI 28-33% (66)
-NRCMS	2008: Ceiling at 20,000 CNY, reimbursement rate of 40% (78)<br>2012: Reimbursement rate for inpatient services at 75% (153)	Lower ceiling than UEBMI (100,000 CNY) and URBMI (80,000 CNY) in 2008 (78)
India:		
-RSBY	No co-payment, but ceiling (12, 24)<br>Inpatient services covered up to an annual ceiling of Rs. 30,000 per family, transport allowance up to Rs. 100/visit with a maximum of Rs. 1,000 per year (24)	Different: ceiling (12)
-Yeshasvini	Co-payment only for outpatient consultations at a network of hospitals (doctors' fees and 30% cost-sharing for diagnostics and tests).<br>Inpatient services covered up to a ceiling of Rs. 100,000 per procedure and Rs. 200,000 per year per person; in addition "pre-authorization" by a resident doctor by the third party administrator required (48, 65)<br>Beneficiaries are only entitled to general ward admission; if they opt for a higher bed category, they have to pay to the hospital (92).	Different (12)
-Rajiv Aarogyasri	No co-payment (154)<br>Inpatient services covered up to a floater of Rs. 150,000 per family per year, in addition Rs. 50,000 as buffer for excess expenses on individual case basis.<br>58% of patients incur OOP average of Rs. 3,600 per patient (67).<br>Annual ceiling of hospital expenses of approx.US$ 3,700 per family (155).	Different (12)
-Kalaignar	No co-payment, but ceiling (12, 24)<br>Inpatient services covered up to a floater of Rs. 100,000 per family for four years (12).	Different (12)
-Vajapayee Arogyasri	No co-payment, but ceiling (12, 24)	n/a

Country and arrangement	Cost-sharing	
	Co-payments, co-insurance, deductibles, ceilings	Same/ different to regularly insured population
Indonesia	No official co-payment (33)	Same for Jamsostek: no cost-sharing<br>Different from Askes: cost-sharing required when services fall outside basic benefit package (33)
Mongolia	Exemption for subsidized population except for nuclear diagnosis and computerized tomography scan, e.g. for children <16 years or <18 if a student, pensioners without any other income source than pension, parents looking after children <2 years, military personnel on active duty, persons specified in Article 18 of social welfare law, handicapped people >50% disability, women with >3 children, etc. (49)	Different<br>For contributors: 5-15% co-payments for inpatient services (10% secondary level, 15% at tertiary level); up to 50% for outpatient essential drugs (88)
Philippines	Reimbursement up to a ceiling for the respective category (especially for inpatient care) (63)<br>Between 10-50% of cost sharing depending on the facility type due to balance billing (63).<br>Hospitalization may require a contribution (35)	n/a
Thailand	Before 2006: 2% or 30 Bath per visit or admission (exemptions for: e.g., older persons, children < 12 years, the poor, etc.)<br>No cost-sharing until 2012<br>Since 2012: 30 Baht co-payment was reintroduced for patients who receive prescriptions and who are willing to pay. Exemptions for: e.g., the poor, older persons, , children < 12 years, etc. (36)	n/a
Vietnam	In case of referral; 5% for the poor and ethnic minorities, 20% for the near-poor and informal sector, higher if referral by-passed<br>Maximum reimbursement for high-tech services of US$ 35 (equal to 40 months of monthly minimum salary)<br>No ceiling for co-payments (15)	Lower for the poor, pensioners, ethnic minorities and those receiving social protection allowance (15)
